# Noise suppression ability and its mechanism analysis of scale-free spiking neural network under white Gaussian noise

**DOI:** 10.1371/journal.pone.0244683

**Published:** 2020-12-31

**Authors:** Lei Guo, Enyu Kan, Youxi Wu, Huan Lv, Guizhi Xu

**Affiliations:** 1 State Key Laboratory of Reliability and Intelligence of Electrical Equipment, School of Electrical Engineering, Hebei University of Technology, Tianjin, China; 2 Hebei Key Laboratory of Bioelectromagnetics and Neuroengineering, Hebei University of Technology, Tianjin, China; 3 School of Artificial Intelligence, Hebei University of Technology, Tianjin, China; Lanzhou University of Technology, CHINA

## Abstract

With the continuous improvement of automation and informatization, the electromagnetic environment has become increasingly complex. Traditional protection methods for electronic systems are facing with serious challenges. Biological nervous system has the self-adaptive advantages under the regulation of the nervous system. It is necessary to explore a new thought on electromagnetic protection by drawing from the self-adaptive advantage of the biological nervous system. In this study, the scale-free spiking neural network (SFSNN) is constructed, in which the Izhikevich neuron model is employed as a node, and the synaptic plasticity model including excitatory and inhibitory synapses is employed as an edge. Under white Gaussian noise, the noise suppression abilities of the SFSNNs with the high average clustering coefficient (ACC) and the SFSNNs with the low ACC are studied comparatively. The noise suppression mechanism of the SFSNN is explored. The experiment results demonstrate that the following. (1) The SFSNN has a certain degree of noise suppression ability, and the SFSNNs with the high ACC have higher noise suppression performance than the SFSNNs with the low ACC. (2) The neural information processing of the SFSNN is the linkage effect of dynamic changes in neuron firing, synaptic weight and topological characteristics. (3) The synaptic plasticity is the intrinsic factor of the noise suppression ability of the SFSNN.

## Introduction

With the development of science and technology, the automation and informatization of human society have been continuously improved, which makes the electromagnetic environment become increasingly complex. Various electromagnetic interference can affect or even damage the operation of the electronic system [1]. The deficiency of traditional protection methods for electronic system including shielding, filtering, grounding and so on become increasingly prominent in the complex electromagnetic environment, which makes electromagnetic protection be faced with increasing serious challenges [2–4]. Biological nervous system has the self-adaptive advantages under the regulation of the nervous system, such as self-learning, self-organizing and self-repairing [5]. It is necessary to explore a new thought on electromagnetic protection by drawing from the self-adaptive advantage of the biological nervous system [6]. Artificial neural network (ANN) is the theoretical and model basis of computational neuroscience, so it is significant to study the robustness of ANN based on brain-like intelligence. The spiking neural network (SNN) is the most biologically interpreted ANN, which can simulate the information processing of the biological brain network by establishing the nonlinear state dynamics behavior of neurons and the regulation process of synaptic weight dynamics [7, 8]. Therefore, an SNN can process complex spatio-temporal information because of its powerful computing capacity [9–11]. SNN can be widely applied in robot control [12], brain-like research [13, 14], pattern recognition [15] and other fields.

The dynamic process of neurons is described by a mathematical model in the form of spiking firing in an SNN. The early integral and fire neuron model are too simple, which is quite different from real neuron characteristics. The H-H model is the fourth-order nonlinear differential equation, which closely represents biological characteristics [16]. The Izhikevich neuron model is the second-order nonlinear differential equation, which not only relatively closely represents real neurons but also has high computing performance [17]. Therefore, most studies are based on the Izhikevich neuron model to construct SNNs. An Izhikevich neuron model was introduced by Nobukawa to evaluate the signal responses of chaotic resonance in SNNs. They confirmed that chaotic states could sensitively respond to weak signals in chaotic resonance [18].

Synaptic plasticity is the basis of information transmission between neurons [19]. The excitability regulation of a synapse can enhance the efficiency of neural information transmission. Most studies are based on excitatory STDP to construct SNNs. A four-pathway excitatory spike-timing dependent plasticity (STDP) rule was proposed by Ebner, who applied the rule to the connection of the pyramidal neuron model, which revealed the interaction between local dynamics of dendritic voltage and plasticity mechanisms [20]. However, biological experiments show that the inhibitory regulation of synapses also plays a vital role in the neural system [21]. Chen et al. [22] used fluorescently tagged gephyrin to track inhibitory synapses in the rat visual cortex and show that visual experience-dependent plasticity is associated with clustered and location-specific of inhibitory synapses. Joana et al. [23] studied the modulation of inhibitory synaptic plasticity on coordinated activity across cortical layers, which found that modulation of inhibitory synaptic strength can effectively influence the participation of cortical neurons to cognition-relevant network activity in the rat barrel cortex. The synaptic plasticity model, including excitability and inhibition synapses, regulates the SNN dynamically, which is more biologically reasonable. The Matthew effect in the network with inhibitory synaptic plasticity showed that good burst synchronization with higher bursting measure improves with long-term potentiation, whereas bad burst synchronization with lower bursting measure becomes worse with long-term inhibition [24]. Research on SNN including both excitatory synapses and inhibitory synapses can reflect the fact that because of change in synaptic strengths, the degree of higher synchronization becomes decreased under the noise of intermediate intensity, while the degree of lower synchronization gets increased under the noise of large intensity [25]. Su et al. [26] studied the regulation process of an SNN under high frequency current stimulation based on synaptic plasticity. It was found that the dynamic behavior of the network is realized by the dynamic weights of synapses, which indicates that synaptic plasticity is the key factor of neurodynamics.

Network topology can reflect the connection form among neurons and affect network functions. A study showed that the distribution of neural connections could affect the propagation of firing rate (FR) and firing pattern in the feed-forward networks [27]. However, an enormous amount of evidence based on fMRI and EEG investigations have suggested that the biological brain function network has a scale-free property and/or small-world property [28, 29]. In our previous work, Zhang et al. found that the rat brain network has the small-world property and that correct working memory storage can increase the connection of the network and efficiency of information transmission [30]. Based on the research results on the biological brain network, an ANN with complex network topology has been studied. Investigations have shown that time delays tuned appropriately can induce multiple stochastic resonances in small-world SNNs based on the WS generation algorithm [31]. Additionally, the influence of STDP on burst synchronization in a scale-free spiking neural network (SFSNN) based on the Barrat Barthelemy Vespignani (BBV) generation algorithm was studied by Kim [32].

The external stimulation can affect the function of the brain network, and the brain network has the self-adaptive response to external stimulation. In the aspect of the study of anti-injury function of robustness in human brain networks, Saeedeh et al. [33] found that human brain networks have a certain degree of anti-injury ability against targeted attack to hub nodes in biological experiment. In the aspect of the anti-injury function in ANN, Nie et al. [34] evaluated the robustness of complex network through the variant of the characteristics (maximal degree, average degree and betweenness), which concluded that the SFN has a certain anti-injury function under node failure or attacks. The researches on the anti-injury function are conducted in the ANNs without nerve electrophysiological characteristics. In other words, the node is not a neuron model and the edge is not a synapse model in the networks. Therefore, this kind of networks cannot receive the external stimulation. The response of the networks without nerve electrophysiological characteristics to external stimulation cannot be studied. SNN is a network with electrophysiological characteristics, so researchers have carried out the researches on the impact of external stimulation on the SNN.

At present, most of the researches on self-adaptive regulation are firing synchronization and neural coding in SNNs under external stimulation. Etémé et al. found that electromagnetic stimulation induces not only regular firing activity of the neuron with spiking and bursting regimes but also synchronous neuronal modes in neural network under magnetic stimulation [35]. In our previous work, the responses of the time coding and the rate coding of the small-world SNN both showed respective specificity under white Gaussian noise and impulse noise [36]. The study of the noise suppression ability of the SNN based on synaptic plasticity is still in the stage of exploration. The research on the robust function of the SNN is of great important for brain science and engineering applications with noise suppression ability based on brain-inspired intelligence. Therefore, we carry out the research on noise suppression ability and its mechanism analysis of SFSNN under white Gaussian noise.

In this study, the SFSNN is constructed and the noise suppression abilities of the SFSNNs with the high average clustering coefficient (ACC) and the SFSNNs with the low ACC are studied comparatively. under white Gaussian noise. Additionally, the noise suppression mechanism of the SFSNN is explored. The main contributions of this study are as follows.

(1) The SFSNN with more biological rationality are constructed. The construction of more brain-like SNNs is an inevitable trend for the development of artificial intelligence.
(2) The noise suppression abilities of the SFSNNs are comparatively analyzed. The results show that the SFSNNs with the high ACC have higher noise suppression performance than the SFSNNs with the low ACC. The result provides a theoretical foundation for the engineering application based on the self-adaptive advantage of the biological nervous system.
(3) The dynamic evolution processing of neuron firing, synaptic weight and topological characteristics is clarified in this study. The result is helpful to understand brain information processing.
(4) The relationship between the external noise suppression ability of the SFSNN and internal synaptic plasticity is established. The result shows that the dynamic regulation of synaptic weight is significantly correlated with the noise suppression ability based on the Pearson correlation coefficient and implies that synaptic plasticity is the intrinsic factor of the noise suppression ability of the SFSNN.

## Methods

### Construction of the SFSNN

The three basic elements of the network construction are the node, edge and topology. In this study, to construct SFSNN, the Izhikevich neuron model is used as a node, which directly affects the information expression. The synaptic plasticity model including excitability and inhibition synapses is used as an edge, which is the basis of information transmission among neurons. The generation algorithm is used to generate the SFNs with a high ACC, which determines the connection form among neurons.

#### Izhikevich neuron model

**Fig 1:**
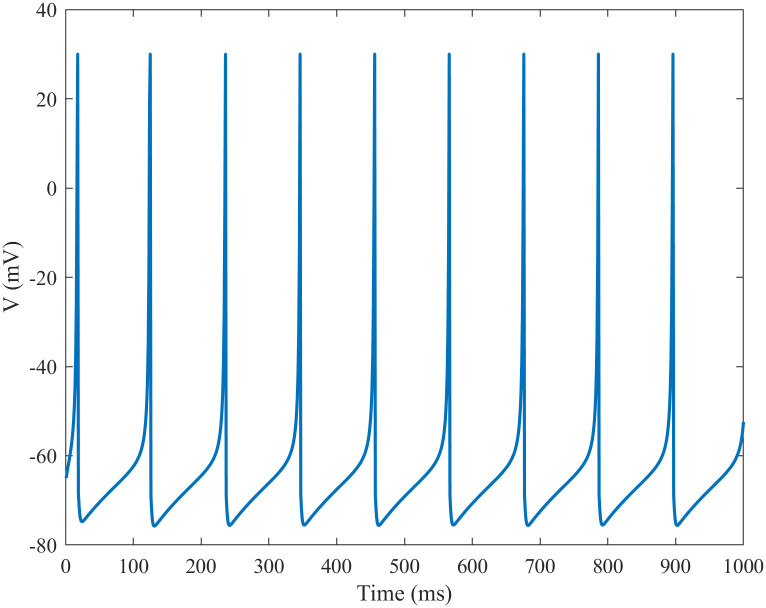
Firing patterns of the regular spiking mode of the Izhikevich neurons. The parameters of the excitatory neuron are: *a* = 0.02, *b* = 0.2, *c* = −65, and *d* = 8.

**Fig 2:**
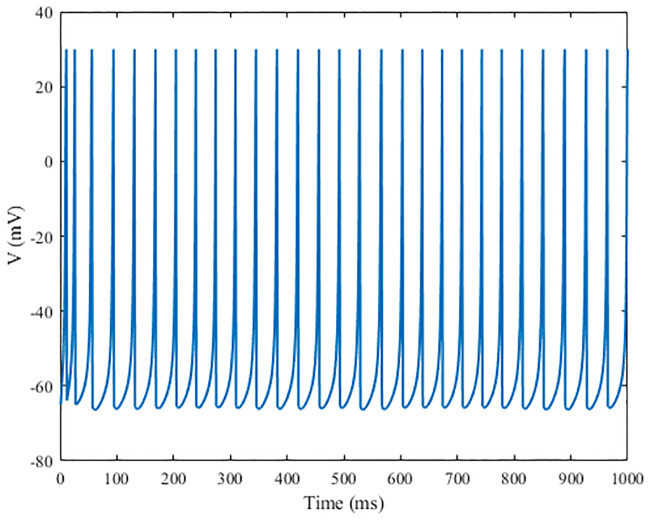
Firing patterns of the low-threshold spiking mode of the Izhikevich neurons. The parameters of the inhibitory neuron are: *a* = 0.02, *b* = 0.25, *c* = −65, and *d* = 2.

The firing characteristics of the Izhikevich neuron model are close to those of real neurons, and it is appropriate for large-scale construction of the network [17]. It is described as:

$$\begin{array}{c} \begin{array}{cc} & \frac{dv}{dt}=0.04v^{2}+5v+140-u+I\text{,}\\ & \frac{du}{dt}=a\left(bv-u\right)\text{,}\\ & if\;v\geq 30\text{,}\;then\{\begin{array}{c} v\leftarrow c\\ u\leftarrow u+d \end{array}\text{,} \end{array} \end{array}$$ (1)

where *v* represents the membrane potential of the neuron, and *u* represents the recovery variable of membrane voltage, which reflects the activation of the potassium channel current and the inactivation of the sodium channel current. And *u* provides negative feedback for the membrane potential *v*. Excitatory and inhibitory neurons are generated by adjusting the dimensionless parameters *a*, *b*, *c*, and *d*. *I* represents the sum of the external input current and synaptic current. Regular spiking pattern is employed as the firing pattern of the excitatory neuron and low-threshold spiking pattern is employed as the firing pattern of the inhibitory neuron, as shown in Figs 1 and 2, respectively.

#### Synaptic plasticity model

The synaptic plasticity model with excitatory synapses and inhibitory synapses plays an important role in regulating the network dynamically. The synaptic output current and input voltage show an approximately linear relationship, which can be described as:

$$\begin{array}{c} I_{syn}={\text{g}}_{syn}\left(t\right)\left(E-V_{j}\left(t\right)\right), \end{array}$$ (2)

where *I*_*syn*_ is the synaptic currents, *g*_*syn*_ is the synaptic conductance, *V*_*j*_(*t*) is the membrane potential of postsynaptic neuron, and *E* is the reversal potential. In this study, the excitatory reversal potential *E*_*ex*_ and the inhibitory reversal potential *E*_*in*_ are 0*mV* and −70*mV*, respectively. Both excitatory and inhibitory synapses regulate the efficiency of information transmission among neurons through the changes of the synaptic conductance, and each synapse has two regulation rules.

(1) When postsynaptic neuron *j* does not receive the action potential of presynaptic neuron *i*, the excitatory synaptic weights and inhibitory synaptic weights are in exponential attenuation. The excitatory and inhibitory synaptic weights are defined as *g*_*ex*_ and *g*_*in*_, respectively, which are described as:

$$\begin{array}{c} \tau_{ex}\frac{d{\text{g}}_{ex}}{dt}=-{\text{g}}_{ex}, \end{array}$$ (3)

$$\begin{array}{c} \tau_{in}\frac{d{\text{g}}_{in}}{dt}=-{\text{g}}_{in}, \end{array}$$ (4)

where *τ*_*ex*_ and *τ*_*in*_ are the attenuation constants of excitatory and inhibitory conductance, respectively [37].

(2) When postsynaptic neuron *j* receives the action potential of the presynaptic neuron *i*, the changes of the excitatory synaptic weight and inhibitory synaptic weight can be described by formulas (5) and (6), respectively.

$$\begin{array}{c} \begin{array}{cc} & {\text{g}}_{ex}\left(t\right)\to {\text{g}}_{ex}\left(t\right)+{\bar{\text{g}}}_{ex}\text{,}\\ & {\bar{\text{g}}}_{ex}\to {w_{ij}}^{*}{\text{g}}_{max}\text{,} \end{array} \end{array}$$ (5)

$$\begin{array}{c} \begin{array}{cc} & {\text{g}}_{in}\left(t\right)\to {\text{g}}_{in}\left(t\right)+{\bar{\text{g}}}_{in}\text{,}\\ & {\bar{\text{g}}}_{in}\to {m_{ij}}^{*}{\text{g}}_{max}\text{,} \end{array} \end{array}$$ (6)

where ${\bar{\text{g}}}_{ex}$ is the excitatory conductance increment caused by the action potential, and it is regulated by synaptic modification function *w*_*ij*_. ${\bar{\text{g}}}_{in}$ is the inhibitory conductance increment caused by the action potential, and it is regulated by synaptic modification function *m*_*ij*_. In this study, *g*_*max*_ is 0.015. When the synaptic weight is less than 0, it is 0. When the synaptic weight is more than *g*_*max*_, it is 0.015. *w*_*ij*_ and *m*_*ij*_ are related to the spiking firing of presynaptic neurons and postsynaptic neurons, respectively, which can be described as:

$$\begin{array}{c} w_{ij}=\{\begin{array}{c} A_{+}\;exp\left(\Delta t/\tau_{+}\right)\text{,}\Delta t<0\\ -A_{-}\;exp\left(-\Delta t/\tau_{-}\right)\text{,}\Delta t\geq 0 \end{array}\text{,} \end{array}$$ (7)

$$\begin{array}{c} m_{ij}=\{\begin{array}{c} -B_{+}\;exp\left(\Delta t/\tau_{+}\right)\text{,}\Delta t<0\\ B_{-}\;exp\left(-\Delta t/\tau_{-}\right)\text{,}\Delta t\geq 0 \end{array}\text{,} \end{array}$$ (8)

where *A*_+_ and *A*_−_ are the maximum modified value of strengthened and weakened synaptic conductivity during excitation process, respectively. *B*_+_ and *B*_−_ are the maximum modified value of strengthened and weakened synaptic conductivity during inhibition process, respectively. Δ*t* is the time interval between presynaptic and postsynaptic neuron firing. *τ*_+_ and *τ*_−_ are the time interval ranges between presynaptic and postsynaptic neuron firing when synapses are strengthened and weakened, respectively.

In this study, the ratio of excitatory synapses to inhibitory synapses is 4:1 in the synaptic plasticity model following the neuroanatomical experiment result of the mammalian cerebral cortex [38]. The parameters are as follows: *τ*_+_ = *τ*_−_ = 20*ms*, *A*_+_ = 0.1, *A*_−_ = 0.105, *B*_+_ = 0.02, and *B*_−_ = 0.03 [39].

#### Generation of the SFN with the high ACC

The BBV generation algorithm is used to generate a weighted SFN in which the topology and network weights can evolve with time. The algorithm steps for network generation are as follows [40]:

(1) Initial network: the network contains *m*_0_ nodes, and the weight *w*_0_ of each edge is 1, where *m*_0_ = 4.
(2) Add new nodes: new node *v* is added with probability *P*, where *P* ∈ (0, 1]. The newly added node has *m* edges, and it is connected with the existing nodes according to priority selection of weight, where *m* = 3. The probability that old node *i* is selected as:
  $$\begin{array}{c} \prod_{v\to i}=\frac{s_{i}}{\sum s_{i}}\;,s_{i}=\sum_{j\in \Gamma (i)}w_{ij}, \end{array}$$ (9)
  where *j* is the node connected to node *i*.
(3) Add new edges: new edges are added according to probability 1 − *P* in the network, only adding *m*_*t*_ edges, where *m*_*t*_ = 2. The two endpoints of the newly added edge are selected according to the triangular mechanism. First, edge (*i*, *j*) of the network is selected randomly, and then another adjacent node *k* of node *j* is selected (excluding node *i*). The probability of selecting node *k* can be calculated as:
  $$\begin{array}{c} \prod_{k}=\frac{w_{jk}}{s_{j}-w_{ij}}. \end{array}$$ (10)

If there is no connection between node *i* and *k*, a new edge is established. Otherwise, the weight is increased by *σ*. In both cases, the weights of edges *w*_*ij*_ and *w*_*jk*_ are increased by *σ*, where *σ* = 1.

In this study, a SFN with the high ACC is generated according to the algorithm above. A SFN is a complex network with the degree distribution following power-law distribution. The probability that a node is connected to other *k* nodes is *P*(*k*) ∼ *k*^−*γ*^. Different SFNs can be obtained by adjusting probability *P*. According to the research results of the functional characteristics of human brain [41], the power-law exponent *γ* is usually in the range of [2, 3], and the clustering coefficient of the SFN is relatively high. Therefore, the SFSNN with a high ACC is constructed, which probability *P* of the SFN is 0.3. The power-law exponent *γ* is 2.15, and the clustering coefficient *C* is 0.5. Among these, clustering coefficient can characterize the aggregation degree of the network. The clustering coefficient of node *i* is defined as the probability that two neighbor nodes of node *i* are connected. It is described as:

$$\begin{array}{c} C_{i}=\frac{2e_{i}}{k_{i}\left(k_{i}-1\right)}, \end{array}$$ (11)

where *k*_*i*_ is the degree of node *i*, *k*_*i*_(*k*_*i*_ − 1)/2 is the possible maximum number of edges, and *e*_*i*_ is the number of edges connected between node *i* and neighbor nodes. The average clustering coefficient of all nodes is used to characterize the clustering coefficient of the network. It is described as:

$$\begin{array}{c} C=\frac{1}{N}\sum_{i=1}^{N}C_{i} \end{array}$$ (12)

**Fig 3:**
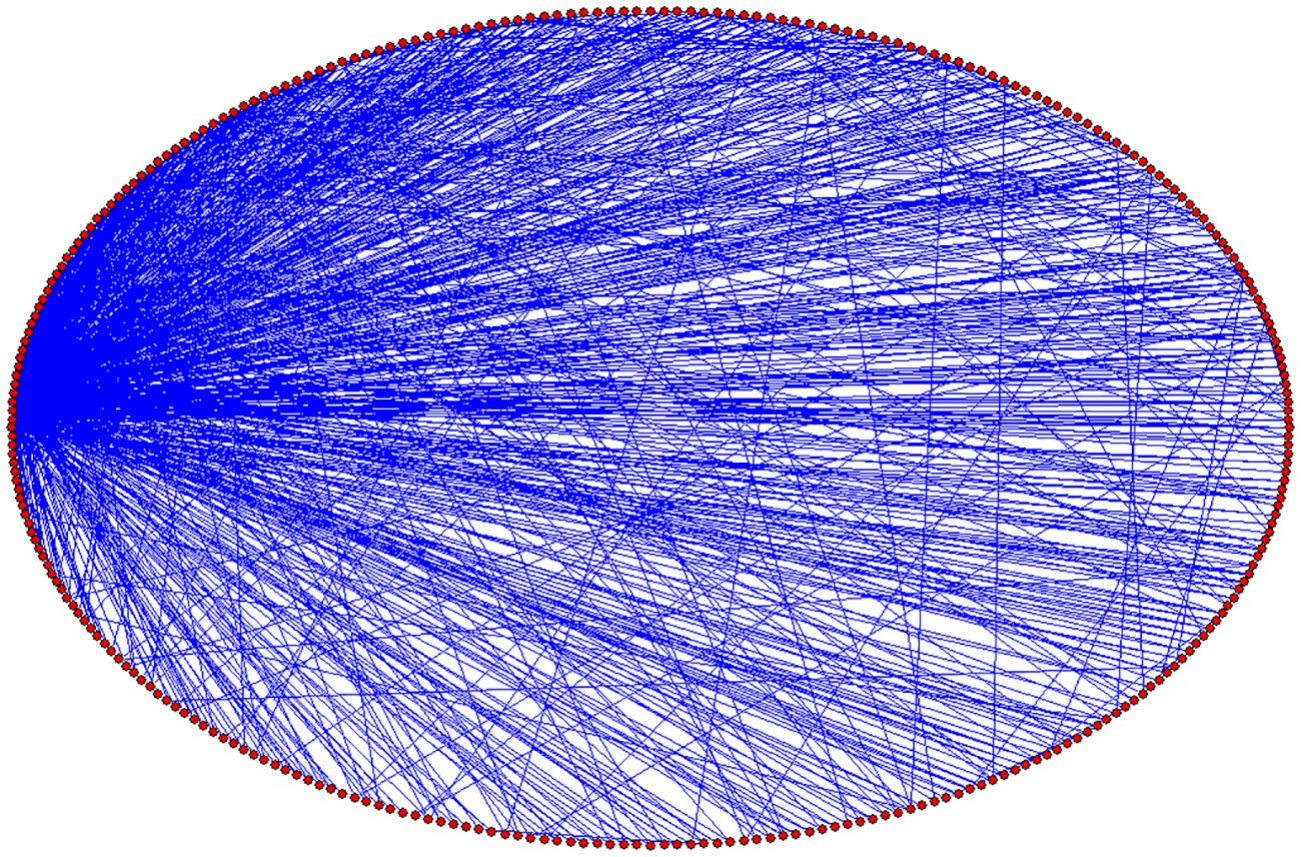
Topological diagram of the SFN. The red dots on the boundary of the ellipse represents 500 nodes, and the internal black line represents the connections between nodes.

**Fig 4:**
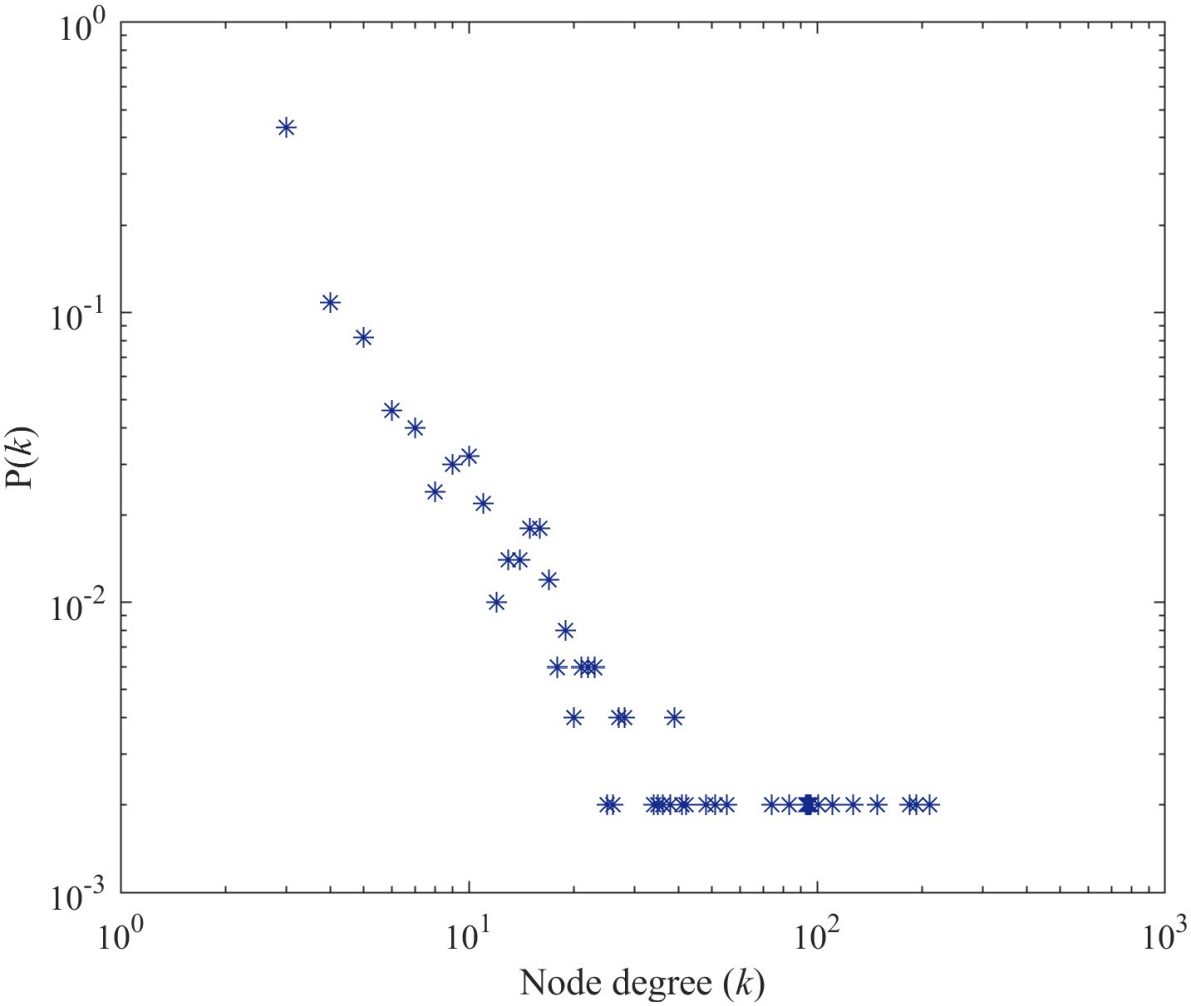
Degree distribution of the SFN. The abscissa represents the node degree value, and the ordinate represents the frequency of the corresponding degree value in the network. The degree distribution of the SFN follows the power-law distribution.

A topological diagram of the SFN is illustrated in Fig 3. The probability distribution of node degree for the SFN when *P* = 0.3 is illustrated in Fig 4.

In this study, to construct SFSNN, the Izhikevich neuron model is used as a node, the synaptic plasticity model is used as an edge, and the BBV generation algorithm is used to generate the SFN. Through the experimental results, the size of the SFSNN is 500 neurons for the following reasons: when the number of neurons of an SFSNN exceeds 1000, a computer cluster needs to be used to compute rather than a single computer due to the computing ability, and we found that noise suppression ability of an SFSNN with the number of neurons in the range of 500-1500 shows no obvious difference. The construction and noise suppression ability analysis of SFSNN are implemented on a PC with a 2.60 GHz CPU and 4 GB RAM.

### Indexes of noise suppression ability

Before and after noise stimulation, the change degree of index is used to evaluate the noise suppression ability of the network. The closer the index of evaluating the noise suppression ability before stimulation to that after stimulation is, the better the noise suppression ability of the network is. In this study, we use two indexes to evaluate the noise suppression ability of the SFSNNs under white Gaussian noise from different angles. One is the relative change rate of the firing rate *δ* which reflects the degree of variation in FR before and after noise stimulation. The other is the correlation coefficient between membrane potentials *ρ* which reflects similarity between membrane potential of neuron before and after noise stimulation.

#### Relative change rate of the firing rate

The interspike interval (ISI) is the difference between two adjacent firing moments of a neuron, which can be calculated as:

$$\begin{array}{c} ISI_{n}=t_{n}-t_{n-1}. \end{array}$$ (13)

where *ISI*_*n*_ is the firing moments difference between the nth neuron and the n-1th neuron, *t*_*n*_ is the firing moment of the nth neuron. In this study, *n* is 500.

In this study, the FR of a neuron is estimated by dividing simulation duration (1000 ms) by the average ISI value. The average FR of all neurons can represent the FR of the SFSNN. The *δ* can quantitatively analyze the degree of variation in the FR before and after white Gaussian noise, which is described as:

$$\begin{array}{c} \delta =\frac{\left|f_{j}-f_{i}\right|}{f_{i}}*100\%, \end{array}$$ (14)

where *f*_*i*_ is the FR before stimulation and *f*_*j*_ is the FR after stimulus, respectively. Under noise stimulation, the smaller the *δ* is, the smaller the degree of change in the FR, and the stronger the noise suppression ability of the SFSNN.

#### Correlation coefficient between membrane potentials

The *ρ* of SFSNN is the average correlation coefficient between membrane potentials of all neurons in the network. The *ρ* can measure the degree of similarity between membrane potentials before and after noise stimulation, which is described as:

$$\begin{array}{c} \rho_{ij}\left(\tau \right)=\frac{\sum_{t=t_{1}}^{t_{2}-\tau +1}x_{i}\left(t\right)x_{j}\left(t+\tau \right)}{\sqrt{\sum_{t=t_{1}}^{t_{2}-\tau +1}{x_{i}}^{2}\left(t\right)\sum_{t=t_{1}}^{t_{2}-\tau +1}{x_{j}}^{2}\left(t+\tau \right)}}, \end{array}$$ (15)

where *ρ*_*ij*_(*τ*) is the correlation coefficient between the neuron membrane potential before and after noise stimulation, *x*_*i*_ is the neuron membrane potential before noise stimulation, *x*_*j*_ is the neuron membrane potential after noise stimulation, [*t*_1_, *t*_2_] is the simulation duration. In this study, the simulation duration is 1000ms. Under noise stimulation, the higher the *ρ* is, the smaller the change in membrane potential, and the stronger the noise suppression ability of the SFSNN.

### The Pearson correlation coefficient

The Pearson correlation coefficient *r* is used to calculate the correlation between the variable *X* and *Y*, which can be described as:

$$\begin{array}{c} r=\frac{\sum_{i=1}^{n}\left(X_{i}-\bar{X}\right)\left(Y_{i}-\bar{Y}\right)}{\sqrt{{\left(X_{i}-\bar{X}\right)}^{2}}\sqrt{{\left(Y_{i}-\bar{Y}\right)}^{2}}}. \end{array}$$ (16)

To determine whether the sample *r* is from the *X* and *Y* related population, it needs to be tested for significance. In this study, we employ the t-test, which is described as:

$$\begin{array}{c} t=\frac{r}{\sqrt{\left(1-r^{2}\right)/\left(n-2\right)}}. \end{array}$$ (17)

When the correlation coefficient is not zero at the significance level of 0.05, it is marked significant with “*” in the top right. When the correlation coefficient is not zero at the significance level of 0.01, it is marked very significant with “**” in the top right. The *P* value is considered statistically significant as follows: “*”means *P* < 0.05, “**” means *P* < 0.01.

## Results and discussion

### Noise suppression ability

White Gaussian noise is the main source of noise for many practical systems, such as radar and communication systems. Therefore, it is currently important to investigate the noise suppression ability of the SFSNN under white Gaussian noise. White Gaussian noise is a kind of noise whose amplitude follows Gaussian distribution, and power spectral density follows uniform distribution. Gaussian distribution function is described as:

$$\begin{array}{c} f\left(x\right)=\frac{1}{\sqrt{2\pi }\sigma }e^{-\frac{{\left(x-\mu \right)}^{2}}{2\sigma^{2}}}. \end{array}$$ (18)

Probability density function of uniform distribution is described as:

$$\begin{array}{c} f\left(x\right)=\{\begin{array}{c} \frac{1}{b-a}\text{,}a<x<b\\ 0\text{,}else \end{array} \end{array}$$ (19)

**Fig 5:**
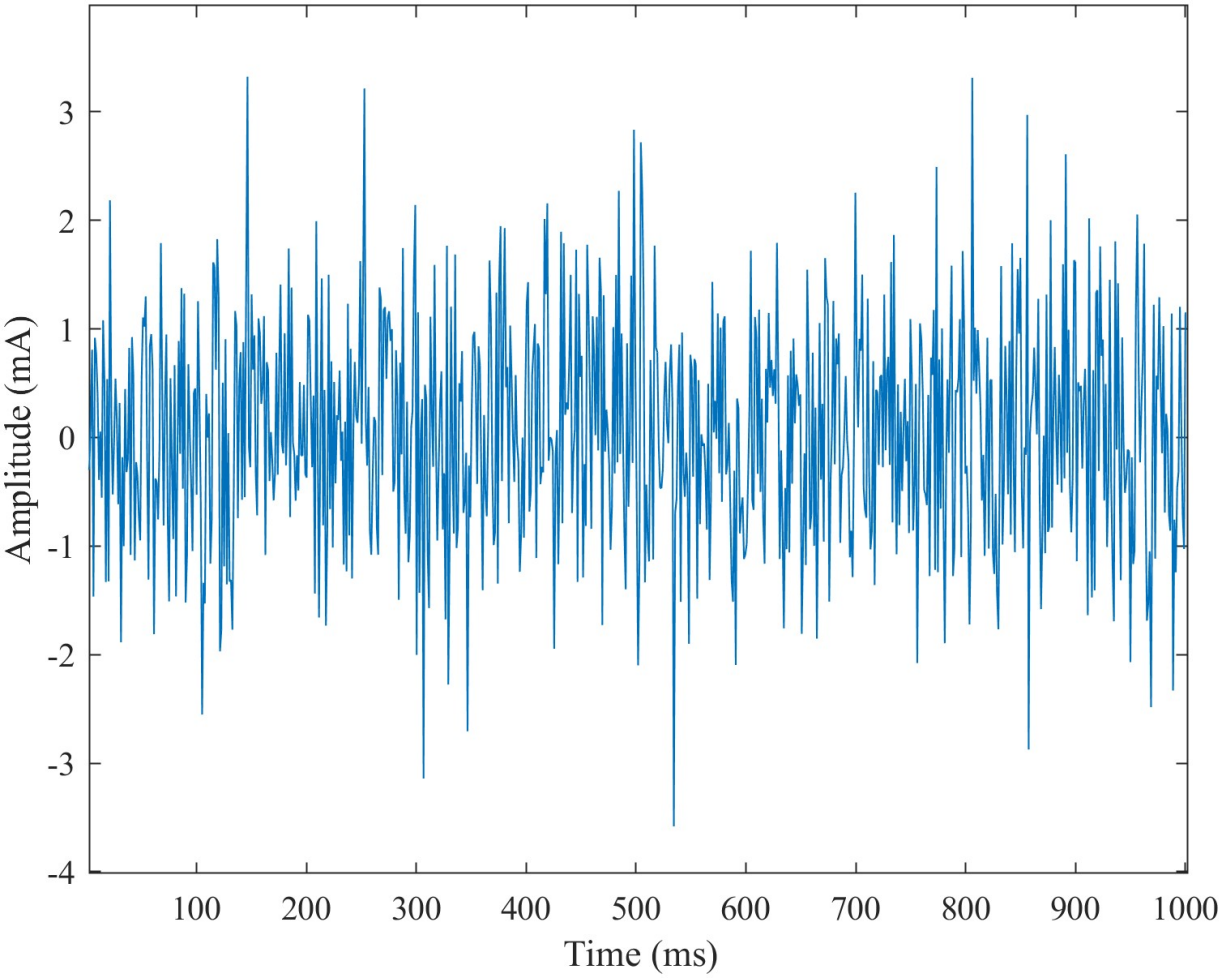
The white Gaussian noise current stimulation.

In this study, white Gaussian noise is the current stimulation. Adding the current stimulation to the neuron model formula (1) can get the neuron model under noise stimulation and then get the SFSNN stimulated by white Gaussian noise. The change of current stimulation amplitude of white Gaussian noise with time as shown in Fig 5.

In this section, *δ* and *ρ* are used as two indexes to evaluate the noise suppression ability of the SFSNNs under white Gaussian noise from different angles. And the noise suppression abilities of the SFSNNs with the high ACC and the SFSNNs with the low ACC are analyzed comparatively and discussed.

#### Noise suppression ability by relative change rate of the firing rate

**Fig 6:**
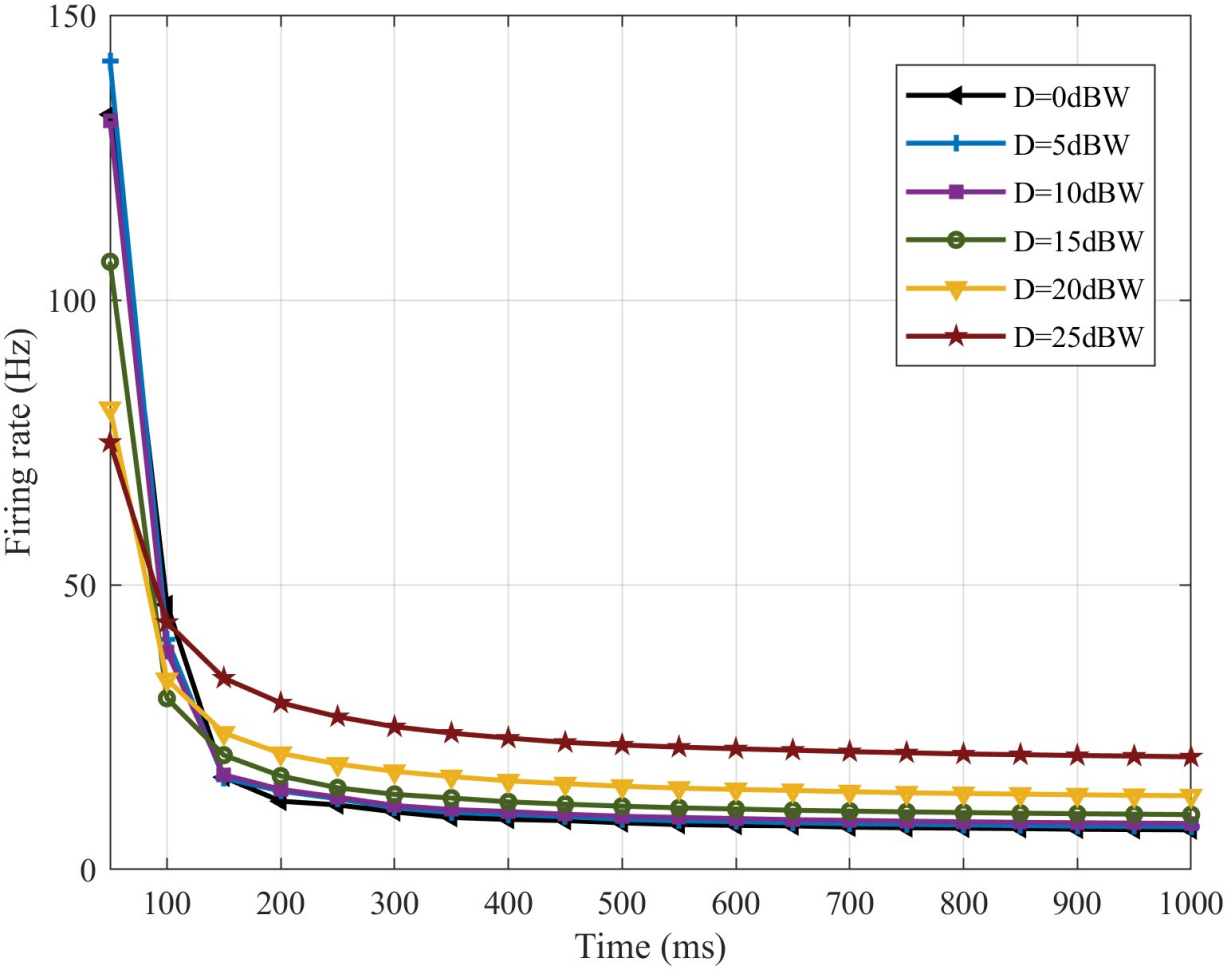
The change in the FR before and after noise stimulation.

To investigate the noise suppression ability of the SFSNN under white Gaussian noise from the angle of *δ*, the change of the FRs before and after noise stimulation is studied according to formula (13). The change of the FRs with time under noise intensities of 0, 5, 10, 15, 20, 25 dBW are illustrated in Fig 6.

From Fig 6, the FR gradually decreases and tends to be stable with time under different intensities of noise. The FR under noise intensities of 5, 10, 15, 20, 25 dBW have small change compared with that before noise stimulation (the noise intensity is 0 dBW), and the degree of the change of the FR gradually increases with the increase of noise intensity. Experiment results show that the SFSNN has a certain degree of noise suppression ability.

**Fig 7:**
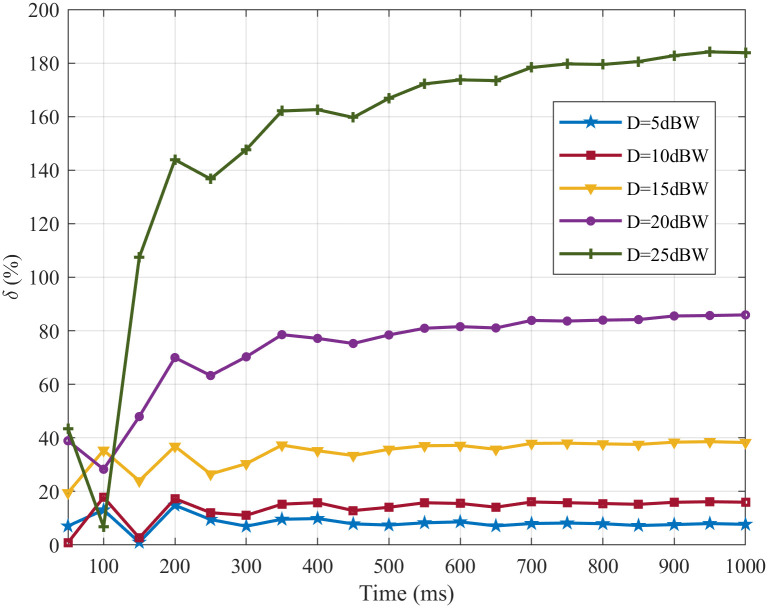
The change in the *δ* with different noise intensities.

To measure the degree of variation in the FR before and after noise stimulation, the change of the *δ* with time under noise intensities of 5, 10, 15, 20, 25 dBW are illustrated in Fig 7.

From Fig 7: with the time, the all of *δ* rise sharply in the first 200 ms, rise slowly from 200 ms to 700 ms and tend to be stable after 700 ms under noise intensities of 5, 10, 15, 20, 25 dBW; when *δ* tend to be stable, the overall trend of the change of *δ* increase with the increase of noise intensity and *δ* are about 7%, 15%, 37%, 84% and 180% under noise intensities of 5, 10, 15, 20, 25 dBW, respectively. The experiment results show that SFSNN has a certain degree of noise suppression ability, and the noise suppression ability becomes weak gradually with the increase of noise intensity from the *δ*.

#### Noise suppression ability by correlation coefficient between membrane potentials

**Fig 8:**
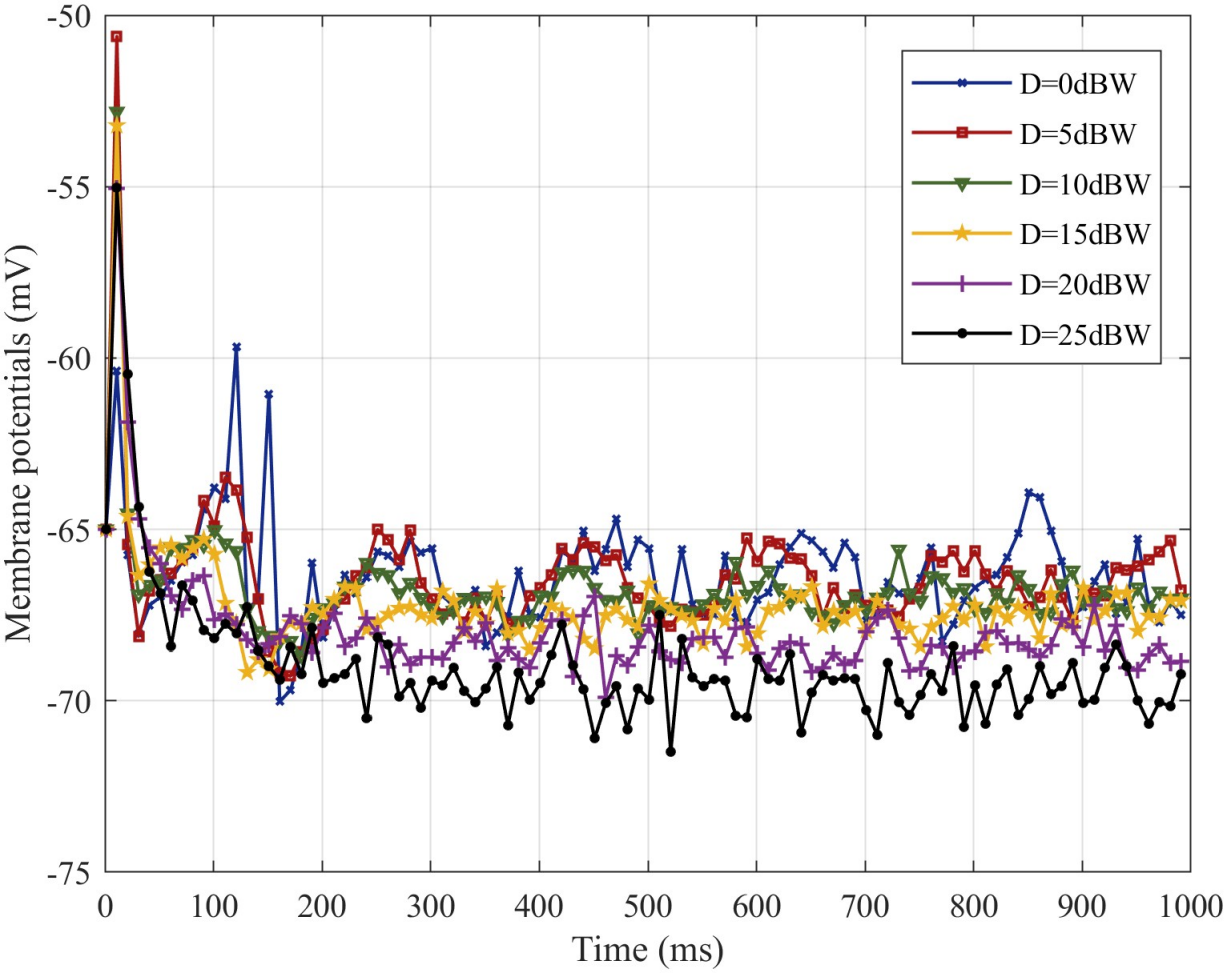
The change in the membrane potentials before and after noise stimulation.

To investigate the noise suppression ability of the SFSNN under white Gaussian noise from the angle of *ρ*, the change of membrane potentials before and after noise stimulation is studied. The change of the membrane potentials with time under noise intensities of 0, 5, 10, 15, 20, 25 dBW are illustrated in Fig 8.

From Fig 8, the membrane potentials gradually decrease and tend to be stable with time under different intensities of noise. The membrane potentials under different intensities of noise have small change compared with the membrane potentials before noise stimulation (the noise intensity is 0 dBW), and the degree of the change of membrane potentials gradually increases with the increase of noise intensity. Experiment results show that the SFSNN has a certain degree of noise suppression ability.

**Fig 9:**
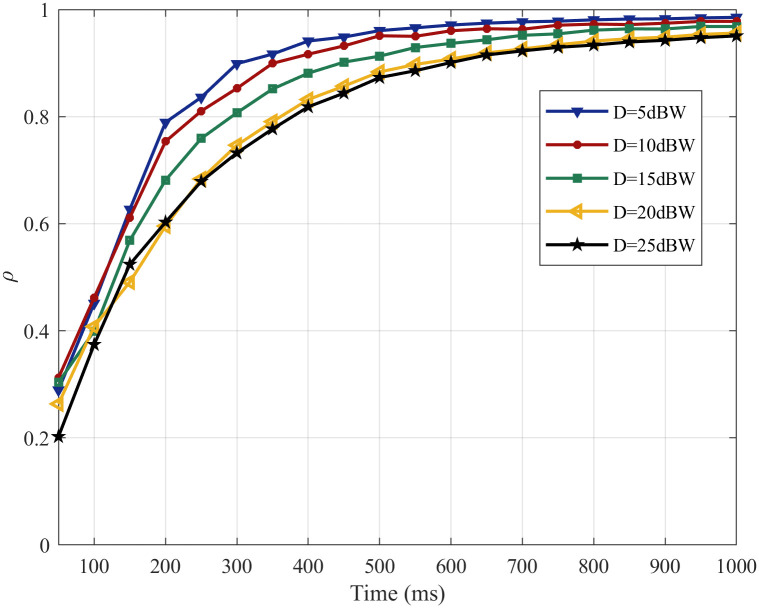
The change in the *ρ* with different noise intensities.

To measure the degree of similarity between membrane potentials before and after noise stimulation quantitatively, the change of the *ρ* with time under noise intensities of 5, 10, 15, 20, 25 dBW are illustrated in Fig 9.

From Fig 9: with the time, the all of *ρ* rise sharply in the first 200 ms, rise slowly from 200 ms to 700 ms and tend to be stable after 700 ms under noise intensities of 5, 10, 15, 20, 25 dBW; when *ρ* tend to be stable, the overall trend of the change of *ρ* increase with the increase of noise intensity and *ρ* are about 0.98, 0.97, 0.96, 0.94 and 0.93 under noise intensities of 5, 10, 15, 20, 25 dBW, respectively. The experiment results show that SFSNN has a certain degree of noise suppression ability, and the noise suppression ability becomes weak gradually with the increase of noise intensity from the *ρ*.

In this study, *δ* and *ρ* are measured to evaluate the noise suppression ability of the SFSNN under white Gaussian noise. *δ* and *ρ* are focused on measuring the degree of variation in FR and similarity between membrane potential of neuron before and after noise stimulation, respectively. Thus, we evaluate the noise suppression ability of the SFSNN from different angles and get the consistent experiment result that the SFSNN has a certain degree of noise suppression ability under white Gaussian noise.

### Comparison of the SFSNNs with the high ACC and the SFSNNs with the low ACC

To investigate the noise suppression abilities of the SFSNNs with the high ACC and the SFSNNs with the low ACC, we attempt to use the BBV algorithm to construct the SFSNNs. However, the ACCs of the SFNs are relatively high and differ slightly based on the BBV algorithm when the power-law exponent is in the range of [2, 3]. Therefore, we can construct the SFSNNs with the high ACC based on BBV algorithm. SFNs with the low ACC is constructed based on the Barabási Albert (BA) generation algorithm [42]. However, the ACCs of the SFNs are relatively low and differ slightly based on the BA algorithm when the power-law exponent is in the range of [2, 3]. Therefore, we can construct the SFSNNs with the low ACC based on BA algorithm.

**Fig 10:**
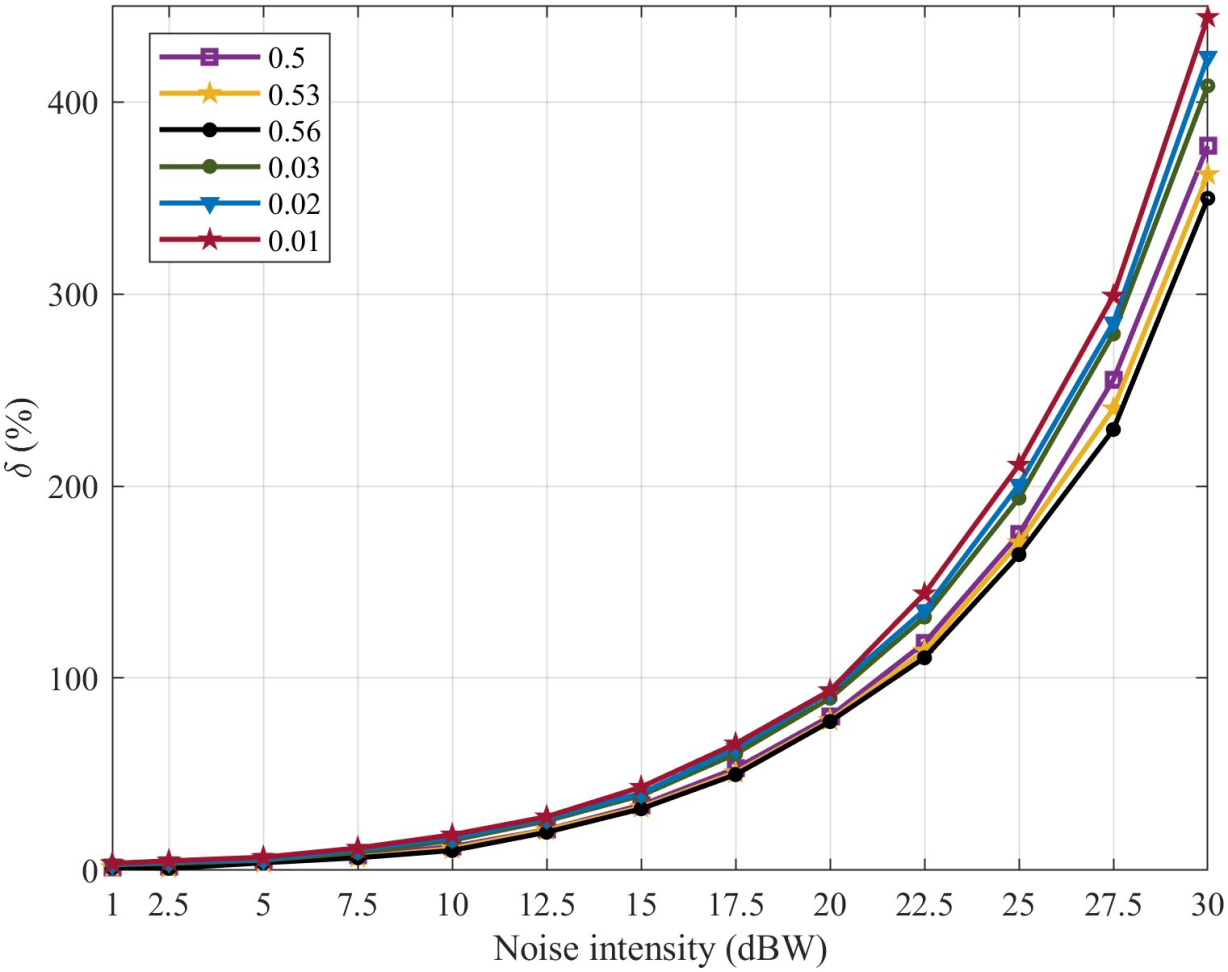
The change of the *δ* of the three SFSNNs with the high ACC and the three SFSNNs with the low ACC.

**Fig 11:**
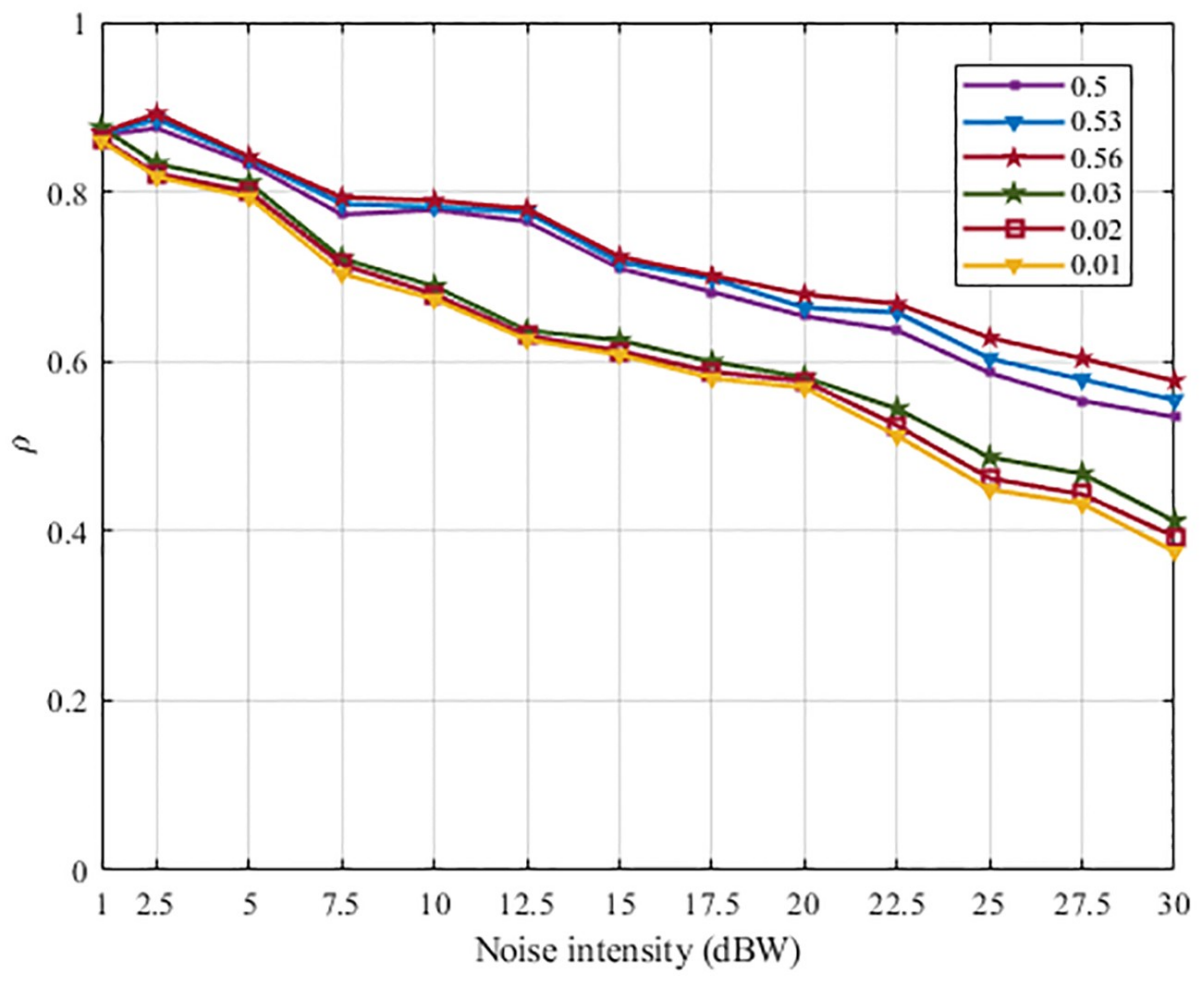
The change of the *ρ* of the three SFSNNs with the high ACC and the three SFSNNs with the low ACC.

In order to comparatively analyze the noise suppression abilities of the SFSNNs with the high ACC and the SFSNNs with the low ACC more statistically, we construct three SFSNNs with the randomly generated high ACC topologies, whose clustering coefficients are 0.50, 0.53 and 0.56, and the corresponding power-law exponents are 2.15, 2.11 and 2.06 based on the BBV algorithm, respectively. And we construct three SFSNNs with the randomly generated low ACC topologies, whose clustering coefficients are 0.01, 0.02 and 0.03, and the corresponding power-law exponents are also 2.24, 2.17 and 2.06 based on the BA algorithm, respectively. The SFSNNs with the low ACC are stimulated under the same noise intensity range of white Gaussian noise. The noise suppression abilities of the SFSNNs with the high ACC are compared with that of the SFSNNs with the low ACC. The noise suppression abilities of the two kinds of SFSNNs are compared by the *δ* and the *ρ*, as illustrated in Figs 10 and 11, respectively.

It can be seen from Figs 10 and 11 that all of the *δ* show an increasing trend, and all of the *ρ* show a decreasing trend in the two SFSNNs with the increase of noise intensity. The *δ* of the three SFSNNs with the randomly generated low ACC topologies are lower than that of the three SFSNNs with the randomly generated high ACC topologies. And the *ρ* of the three SFSNNs with the randomly generated high ACC topologies are higher than that of the three SFSNNs with the randomly generated low ACC topologies. Therefore, from two indexes *δ* and *ρ* of evaluating noise suppression ability, we get the consistent experiment result that the SFSNNs with the high ACC have higher noise suppression performance than the SFSNNs with the low ACC on the whole.

**Fig 12:**
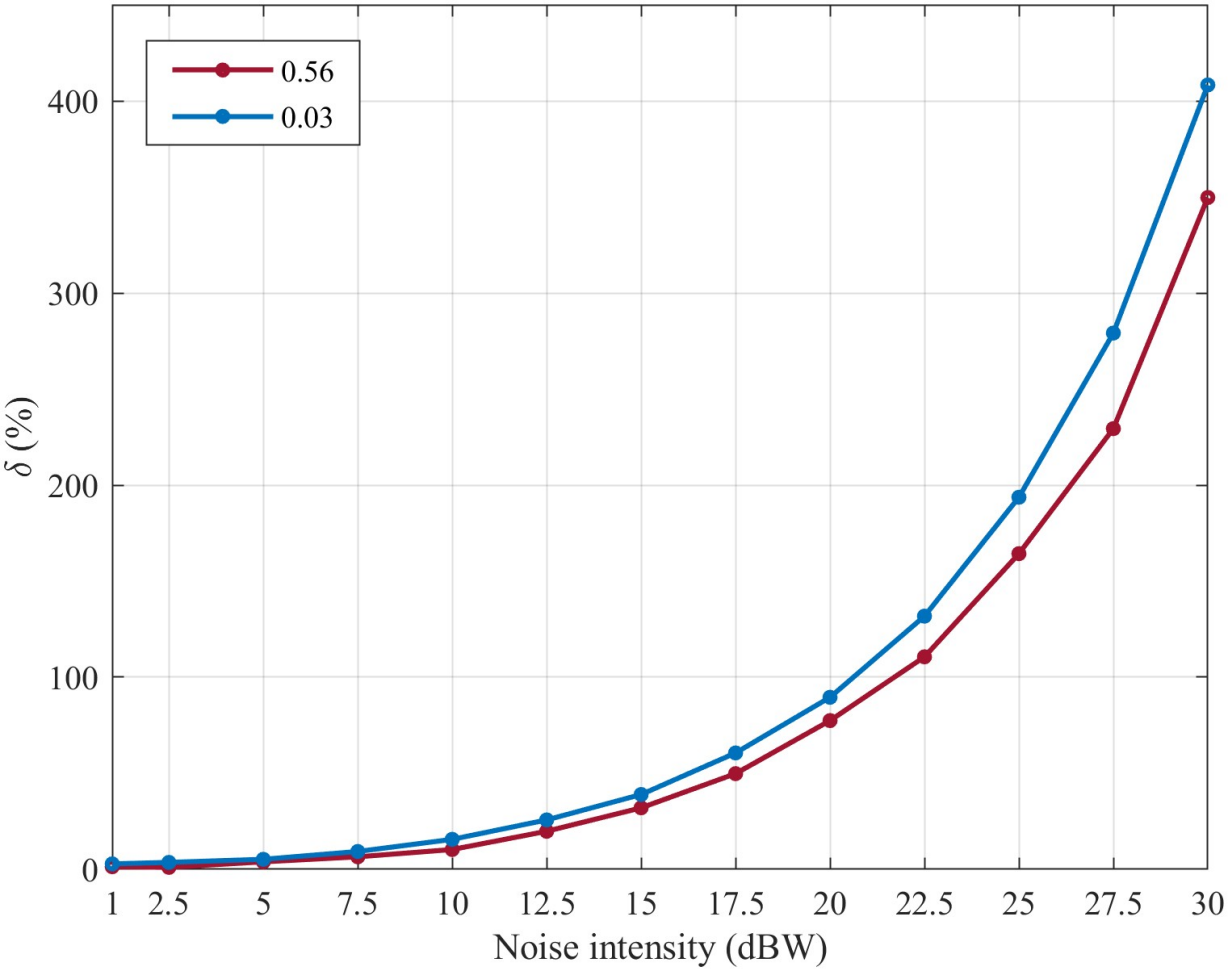
The change of the *δ* of a SFSNN with the high ACC and a SFSNN with the low ACC.

**Fig 13:**
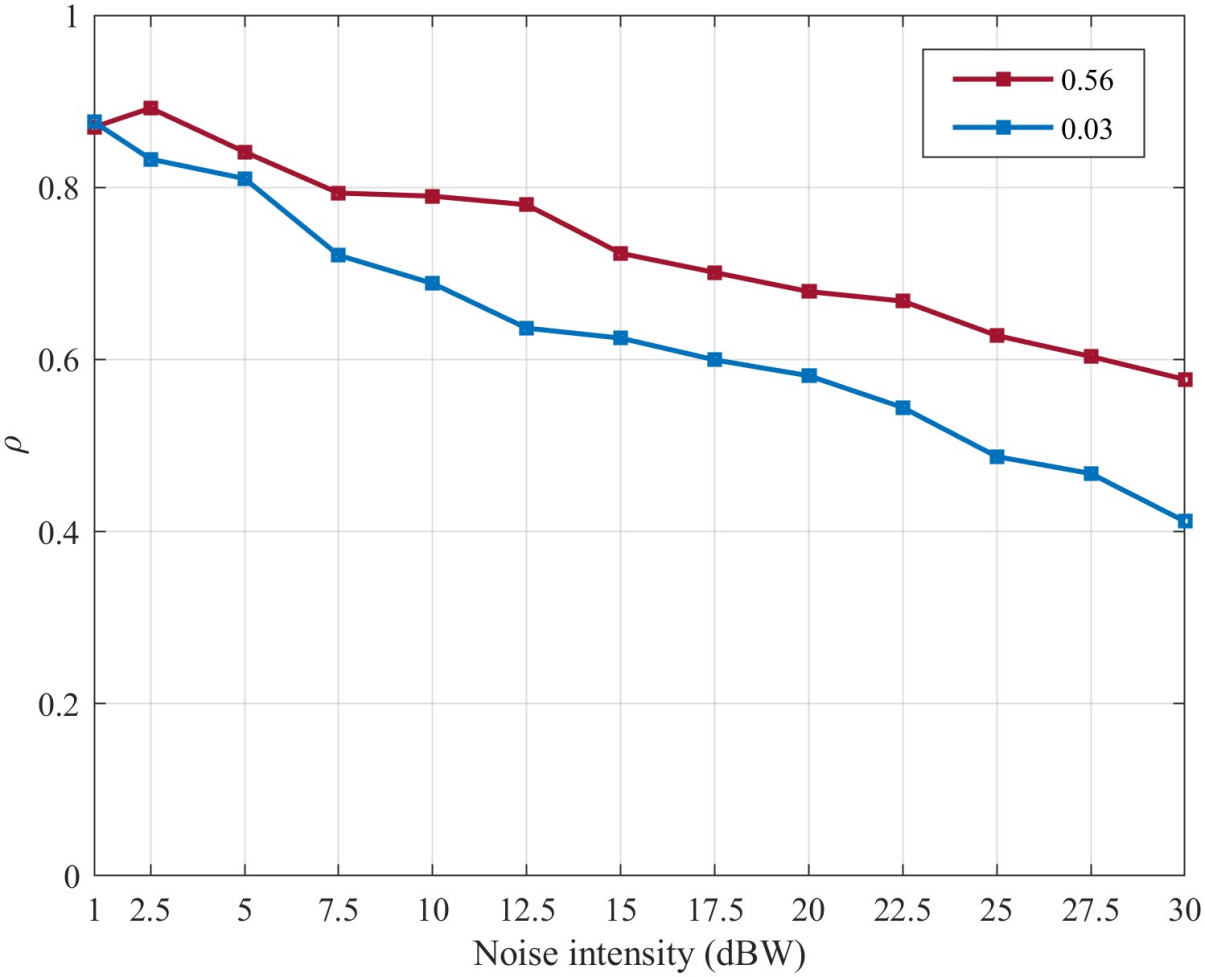
The change of the *ρ* of a SFSNN with the high ACC and a SFSNN with the low ACC.

To quantitatively analyze the difference in the noise suppression abilities of the SFSNNs with the high ACC and the SFSNNs with the low ACC, the Euclidean distance is used to calculate the difference of the *δ* between the SFSNN with the high ACC of 0.56 and the SFSNN with the low ACC of 0.03, the result is 87.25 and illustration is shown in Fig 12; the Euclidean distance of the *ρ* between above these two SFSNNs is calculated, the result is 0.39 and illustration is shown in Fig 13.

To eliminate the dimensional difference, the Euclidean distance of the *δ* and the *ρ* between the above two kinds of SFSNNs are recalculated through data normalization and are 0.03 and 0.32, respectively. The experiment results show that the SFSNNs with the high ACC have higher noise suppression performance than the SFSNNs with the low ACC.

### Noise suppression mechanism analysis of the SFSNN

To explore the noise suppression mechanism of the SFSNN, we analyze intrinsic factor of the noise suppression ability from the two aspects. (1) The dynamic evolution process of the information processing of the SFSNN under white Gaussian noise. (2) The relationship between the external noise suppression ability of the SFSNN and internal synaptic plasticity.

#### Neural information processing of the SFSNN

To explore the neural information processing of the SFSNN, the dynamic evolution processes of the firing rate, synaptic weight and clustering coefficient under white Gaussian noise of 10 dBW are investigated.

(1) Firing Rate

**Fig 14:**
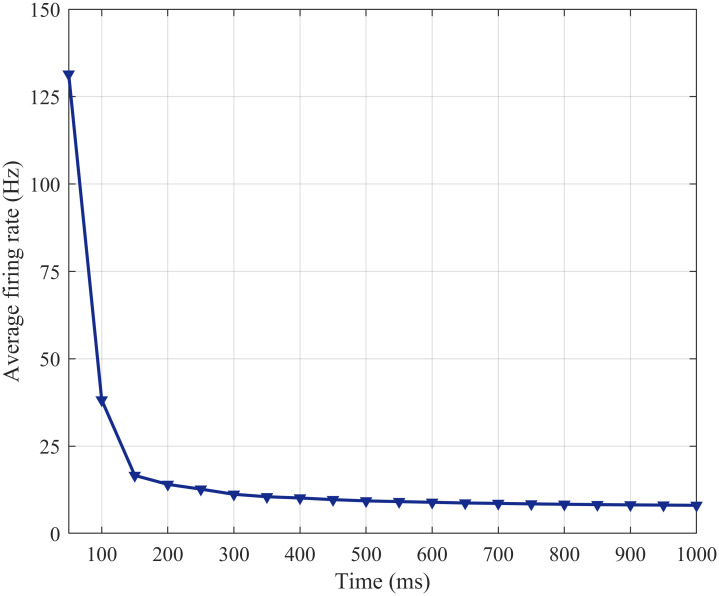
The change in the average FR with time.

The external white Gaussian noise can lead to the change of the firing sequence of neurons in the network. To describe the FR of all neurons of the SFSNN, the average FR is used, and its dynamic evolution is illustrated in Fig 14.

From Fig 14, the average FR drops sharply in the first 150 ms, drops slowly from 150 ms to 700 ms and tends to be stable after 700 ms under white Gaussian noise of 10 dBW. And the firing moment of neurons is an important factor in the change in synaptic weight.

(2) Synaptic Weight

**Fig 15:**
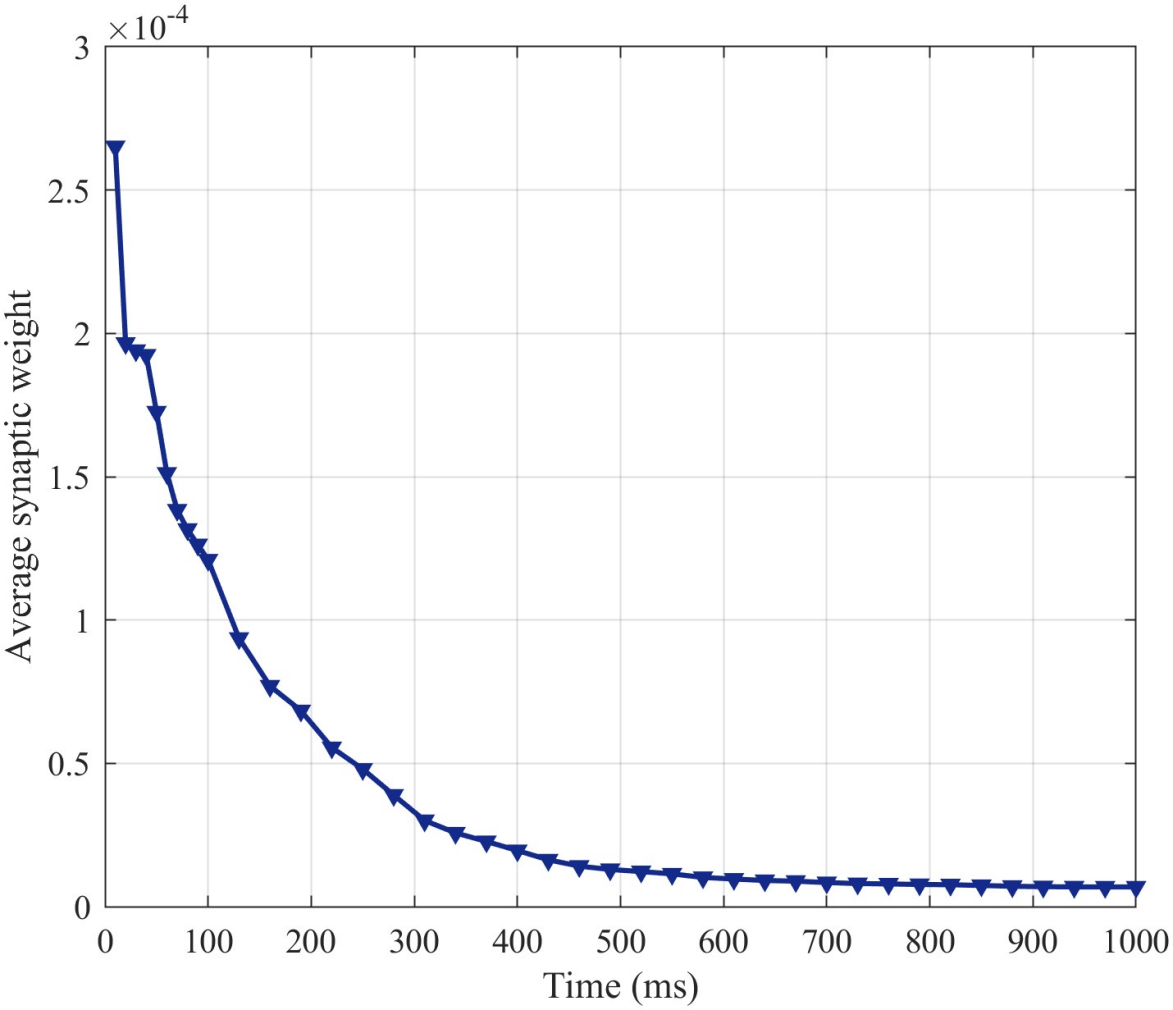
The change in the average synaptic weight with time.

According to formulas (3), (4), (5), (6), (7) and (8), it can be found that the noise stimulation can affect the firing moments interval between presynaptic and postsynaptic neurons Δ*t*, and the weight of excitatory synapses *g*_*ex*_(*t*) and the weight of inhibitory synapses *g*_*in*_(*t*) is affected by Δ*t*. Therefore, the change of the FR can lead to the change of synaptic weight. The average synaptic weight can be used to describe the synaptic weight of all edges in the SFSNN. The dynamic evolution of the average synaptic weight with time under the noise is illustrated in Fig 15.

From Fig 15, the average synaptic weight drops obviously in the first 150 ms and decreases slowly from 150 ms to 700 ms; the average synaptic weight tends to be stable after 700 ms.

**Fig 16:**
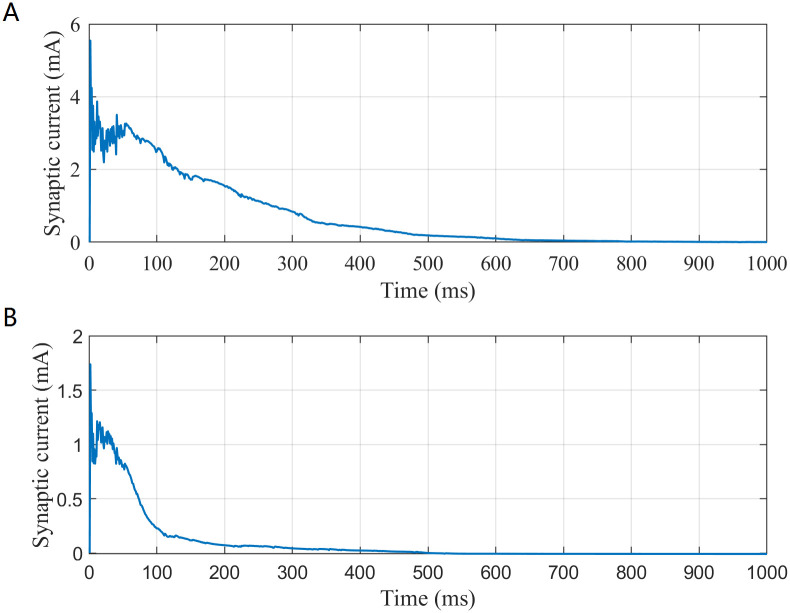
The change in the synaptic current with time. (A) The excitatory current. (B) The inhibitory current.

And according to formula (2), it can be found that the change of synaptic weight *g*_*syn*_ can change synaptic currents *I*_*syn*_. And the synaptic currents include excitatory and inhibitory currents. In this study, excitatory synaptic current is the mean of excitatory current received by all postsynaptic neurons; inhibitory synaptic current is the mean of inhibitory current received by all postsynaptic neurons. To investigate the change in the inhibitory and excitatory synaptic currents with time under white Gaussian noise, their dynamic evolution are illustrated in Fig 16.

From Fig 16, the excitatory current drops sharply in the first 200 ms, drops slowly from 200 ms to 700 ms and tends to be stable after 700 ms. The inhibitory current drops sharply in the first 100 ms, drops slowly from 100 ms to 200 ms and tends to be stable after 200 ms. Because of the influence of synaptic dynamic regulation on synaptic current, excitatory and inhibitory current also gradually decreases and tends to be stable during the process of regulation. From the experiment results, synaptic plasticity plays a role in regulating the SFSNN under white Gaussian noise. During the process of regulation, the synaptic weight gradually decreases and tends to be stable.

(3) Clustering Coefficient

**Fig 17:**
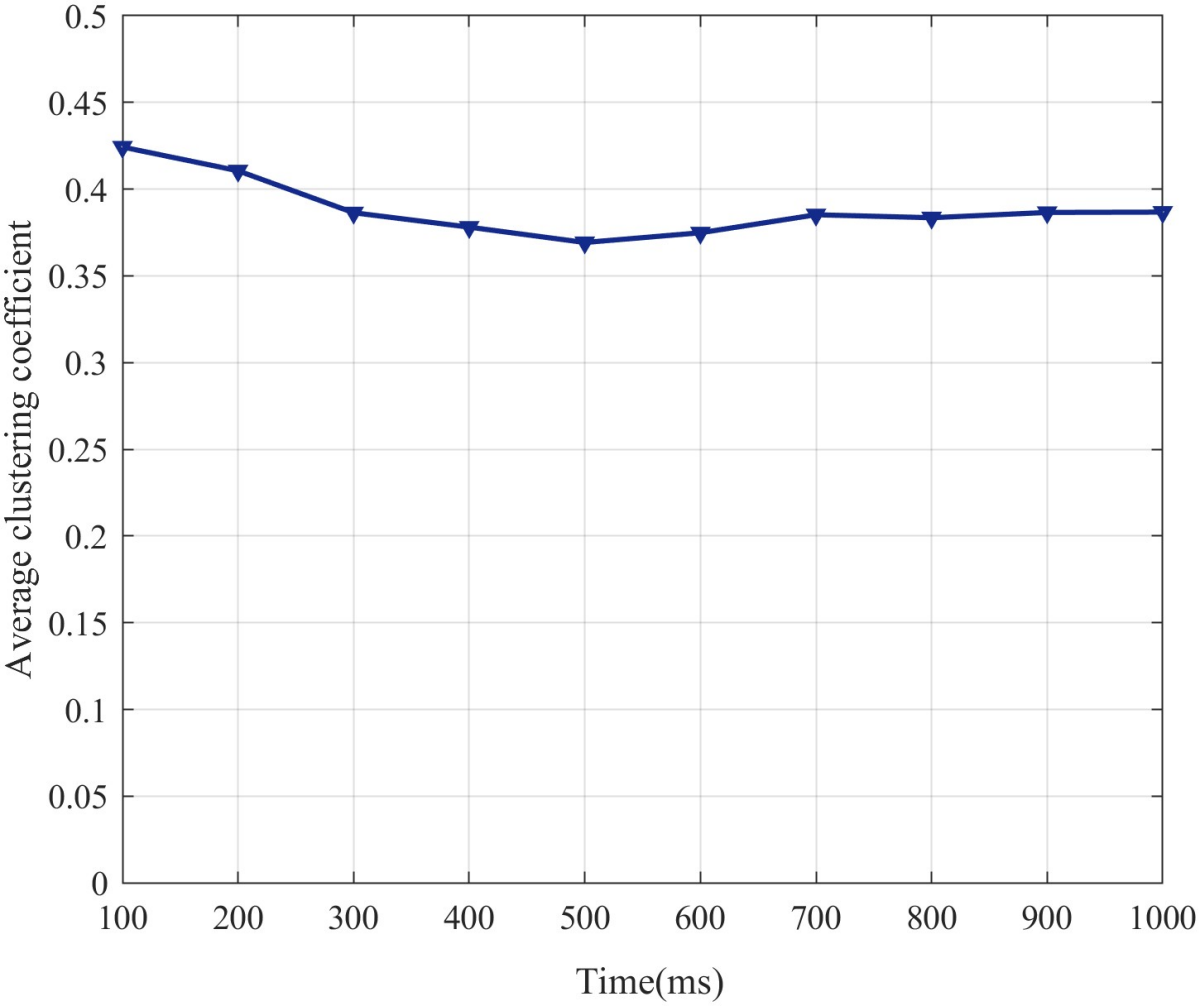
The change in the ACC with time.

As an important index to measure the topological characteristics of an SFSNN, the clustering coefficient reflects local information transmission efficiency of the network. For a weighted network, the clustering coefficient of the node *i* is described as [43]:

$$\begin{array}{c} c_{i}=\frac{1}{s_{i}\left(k_{i}-1\right)}\sum_{j,k}\frac{\left(g_{ij}+g_{ik}\right)}{2}a_{ij}a_{jk}a_{ki} \end{array}$$ (20)

where *g*_*ij*_, *g*_*ik*_ are the synaptic weight of weighted network W and *k*_*i*_, *s*_*i*_ are the degree and strength of the node i, respectively. In this study, the SFSNN is a network with dynamic regulation of synaptic weight. And according to formula (20), we can found that the change of synaptic weight *g* can lead to the change of the clustering coefficient *C*_*i*_. Therefore, the dynamic regulation of the synaptic weight forms synaptic plasticity and it also can change the topological structure of the SFSNN. The ACC can describe the clustering coefficient of all neurons in the SFSNN, and dynamic evolution of the ACC with time under white Gaussian noise is illustrated in Fig 17.

From Fig 17, the ACC decreases gradually within 450 ms, increases gradually from 450 ms to 700 ms and becomes stable from 700 ms to 1000 ms.

From the theory and experiment results, white Gaussian noise can lead to the change of the firing sequence of neurons in the network. Synaptic plasticity regulates synaptic weight through the change of firing moment of presynaptic neurons and postsynaptic neurons. Therefore, the change of the FR can lead to the change of the synaptic weight. In this study, the SFSNN is a network with dynamic regulation of synaptic weight. Therefore, the dynamic regulation of the synaptic weight also can change the topological structure of the SFSNN. The experiment results show that the dynamic evolution of neural information processing presents a trend from intense to gradually stable. Furthermore, the neural information processing of the SFSNN is the linkage effect of dynamic evolution in neuron firing, synaptic weight and topological structure.

#### Correlation analysis based on the Pearson correlation coefficient

To further explore the noise suppression mechanism of the SFSNN, the relationship between the external noise suppression ability of the SFSNN and the internal synaptic plasticity is established. An analysis of the correlation between the synaptic plasticity and the noise suppression ability of the SFSNN is conducted based on the Pearson correlation coefficient. To analyze the correlation between the dynamic regulation of synaptic weight and the *δ*, and the correlation between the dynamic regulation of synaptic weight and the *ρ*, the Pearson correlation coefficient is calculated according to formula (16) and (17). In this study, *X* represents the average synaptic weight in every 50 ms; *Y* represents the average FR or the average *ρ* in every 50 ms; and *n* represents the numbers of *X* and *Y*. The simulation time is 1000 ms, and the time interval is 50 ms. Thus, *n* is 20.

The correlation coefficient between the average synaptic weight and the *δ* is -0.961** (*P* < 0.01), and the correlation coefficient between the average synaptic weight and the *ρ* is -0.995** (*P* < 0.01), which shows that the dynamic regulation of synaptic weight is significantly correlated with the noise suppression ability of the SFSNN at the significance level of 0.01 (two-sided t-tests). The above results imply that synaptic plasticity is the intrinsic factor of the noise suppression ability of the SFSNN.

## Conclusion

In this study, the SFSNN with more biological rationality is constructed to study the noise suppression ability under white Gaussian noise. Furthermore, the neural information processing of the SFSNN is investigated, and the noise suppression mechanism of the SFSNN is explored. The experiment results indicate the following. (1) We evaluate the noise suppression ability of the SFSNN from different angles and get the consistent experiment result that the SFSNN has a certain degree of noise suppression ability under white Gaussian noise. (2) The *δ* of the SFSNN with the high ACC are lower than that of the SFSNN with the low ACC, whereas the *ρ* of the SFSNN with the high ACC are higher than that of the SFSNN with the low ACC. The result shows that the SFSNN with the high ACC have higher noise suppression performance than the SFSNN with the low ACC on the whole. (3) The neural information processing of the SFSNN is the linkage effect of dynamic changes in neuron firing, synaptic weight and topological structure. (4) The dynamic regulation of synaptic weight is significantly correlated with the noise suppression ability, which shows that synaptic plasticity is the intrinsic factor of the noise suppression ability of the SFSNN. This study can be helpful to understand the brain information processing and provides theoretical foundation for the engineering application of robustness drawing from the self-adaptive advantage of the biological nervous system.

The ANNs without nerve electrophysiological characteristics cannot receive the external stimulation, in which the node is not a neuron model and the edge is not a synapse model. Therefore, the response of this kind of networks to external stimulation cannot be studied. For the SNNs, most of the researches on self-adaptive regulation are firing synchronization and neural coding under external stimulation. The study of the noise suppression ability of the SNN based on synaptic plasticity is still in the stage of exploration. This study can be helpful to understand the brain information processing under external stimulation and provides theoretical foundation for the engineering application of robustness drawing from the self-adaptive advantage of the biological nervous system.

## Supporting information

S1 File Data required for all study findings reported in the article. (ZIP)
